# Plasma Levels and Diagnostic Utility of VEGF in a Three-Year Follow-Up of Patients with Breast Cancer

**DOI:** 10.3390/jcm10225452

**Published:** 2021-11-22

**Authors:** Grażyna E. Będkowska, Ewa Gacuta, Monika Zbucka-Krętowska, Paweł Ławicki, Maciej Szmitkowski, Adam Lemancewicz, Joanna Motyka, Agnieszka Kobus, Monika Chorąży, Marlena Paniczko, Sławomir Ławicki

**Affiliations:** 1 Department of Haematological Diagnostics, Medical University of Bialystok, ul. Waszyngtona 15A, 15-269 Bialystok, Poland; grazyna.bedkowska@umb.edu.pl; 2 Department of Perinatology, Medical University of Bialystok, ul. M. Słodowskiej-Curie 24A, 15-276 Bialystok, Poland; sunnyeve@wp.pl (E.G.); adam.lemancewicz@umb.edu.pl (A.L.); 3 Department of Gynecological Endocrinology and Adolescent Gynecology, Medical University of Bialystok, ul. M. Słodowskiej-Curie 24A, 15-276 Bialystok, Poland; monikazbucka@wp.pl; 4 Department of Population Medicine and Lifestyle Diseases Prevention, Medical University of Bialystok, ul. Waszyngtona 13A, 15-269 Bialystok, Poland; pawellawicki04@gmail.com (P.Ł.); joanna.motyka@umb.edu.pl (J.M.); marlena.paniczko@umb.edu.pl (M.P.); 5 Department of Biochemical Diagnostics, Medical University of Bialystok, ul. Waszyngtona 15A, 15-269 Bialystok, Poland; msz@umb.edu.pl; 6 Department of Dentistry Propaedeutics, Medical University of Bialystok, ul. Szpitalna 30, 15-295 Bialystok, Poland; agnieszka.kobus@umb.edu.pl; 7 Department of Neurology, Medical University of Bialystok, ul. M. Słodowskiej-Curie 24A, 15-276 Bialystok, Poland; chorazym@op.pl

**Keywords:** breast cancer, VEGF, CA 15-3, tumor marker

## Abstract

Breast cancer is the most common malignancy in women globally. The increasing worldwide incidence of this type of cancer illustrates the challenge it represents for healthcare providers. Therefore, new tumor markers are constantly being sought. The aim of this study was to assess plasma concentrations and the diagnostic power of VEGF in 100 patients with early-stage breast cancer, both before and after surgical treatment and during a three-year follow-up. The control groups included 50 subjects with benign breast tumors (*fibroadenoma*) and 50 healthy women. The VEGF concentration was determined using enzyme-linked immunosorbent assay (ELISA) and the CA 15-3 concentration was determined by chemiluminescent microparticle immunoassay (CMIA). We observed significantly higher preoperative plasma concentrations of VEGF and CA 15-3 in patients with breast cancer. VEGF, similar to CA 15-3, demonstrated high diagnostic utility in the assessment of the long-term efficacy of surgical removal of the tumor. Determinations of VEGF had the highest diagnostic usefulness in the detection of breast cancer recurrence (SE 40%, SP 92%, PPV 67%, NPV 79%). Additionally, the highest values of SE, NPV and AUC were observed during the combined analysis with CA 15-3 (60%; 84%; 0.7074, respectively). Our study suggests a promising diagnostic utility of VEGF in the early stages of breast cancer and in the evaluation of the efficacy of the surgical treatment of breast cancer as well as the detection of breast cancer recurrence, particularly in a combined analysis with CA 15-3 as a new diagnostic panel.

## 1. Introduction

Breast cancer (BC) is the most common malignant neoplasm in women and a major global problem. In 2018, more than 2 million new cases and over 620,000 deaths were reported. The clinical course of BC varies considerably between patients, as does the response to treatment [1,2]. The early detection of neoplastic lesions as well as recurrent and metastatic cancer is crucial to cancer outcomes. Significant improvements in tumor detection have been achieved due to the application of imaging techniques, including ultrasound and magnetic resonance imaging (MRI), and biochemical tests. The American Society of Clinical Oncology guidelines include markers such as the estrogen receptor (ER), progesterone receptor (PR) and human epidermal growth factor receptor 2 (HER2) as useful and non-invasive tests that can help determine the prognosis and further treatment, and markers such as CEA and CA15-3 in the monitoring of treatment in BC. However, the effectiveness of imaging modalities in detecting small neoplastic lesions is limited and the utility of biomarkers is still a subject of debate within the scientific community [3,4,5].

There are two main stages in the development of every neoplastic lesion: the growth of the primary tumor and metastasis [6]. The development of both is controlled and regulated by the processes of angiogenesis and lymphangiogenesis, which depend on the proportion between negative and positive endothelial regulators [7,8]. Numerous reports have demonstrated that the development of BC results in the increased activity of many factors that contribute to the severity of both processes [9,10]; vascular endothelial growth factor (VEGF) stands out as the main angiogenic factor in malignant tumors [11]. VEGF exerts a specific effect on endothelial cells not only by stimulating cell growth, but also by impacting their migration and vascular permeability [12,13]. Moreover, our own studies [14,15,16] and those of other researchers [17,18,19] have shown an increased expression of VEGF in BC, suggesting its possible prognostic utility. Therefore, new diagnostic methods and markers that are capable of detecting neoplastic lesions as early as possible are still being sought. We believe that VEGF holds promise for becoming such a marker.

The aim of this study was to evaluate the concentrations of VEGF and CA 15-3 in the plasma of patients with early-stage BC and in control groups, which consisted of a group of patients with benign breast lesions (*fibroadenoma*) and a group of healthy individuals. Furthermore, the utility of the studied parameters in the assessment of the long-term efficacy of the surgical removal of BC, as well as in the detection of recurrence, was assessed via a three-year follow-up.

## 2. Materials and Methods

Characteristics of the study and control groups are presented in Table 1. The current, prospective study included 100 patients with BC (*ductal adenocarcinoma*) who underwent surgery and received adjuvant therapy at Bialystok Cancer Centre (Poland). The inclusion criteria for these patients were: complete clinicopathological data including age, menopausal status, race (Caucasian) and cancer risk factors; the clinical stage, size and histopathological type of the tumor; axillary lymph node status; expression of ER, PR and Ki-67. Tumors were classified and their stage was determined in accordance with the International Union against Cancer Tumor-Node-Metastasis (UICC-TNM) classification. Histopathology of BC was assessed in all cases by a preoperative biopsy of the mammary tumor or from the intraoperatively obtained tumor tissue samples (all patients with *adenocarcinoma ductale*). Patients were divided into groups depending on the cancer stage, which was established using the TNM system (I and II).

The control group consisted of 50 subjects with benign breast lesions (*fibroadenoma*), who also underwent surgical treatment, and 50 healthy women. Patients with fibroadenomas were chosen as a control group because although they underwent surgical treatment after the menopause, their lesions were diagnosed when the patients were still premenopausal. In the period between the diagnosis and surgery, the patients remained in the care of oncologists for around five years. The inclusion criteria for these subjects were also the complete clinicopathological data including cancer risk factors (family history), race (Caucasian), age, menopausal status and the histopathological type of the lesion, which was pathologically confirmed by prior core needle biopsy. The selection of the study and control groups, the pre- and postoperative therapeutic management and the administration of adjuvant therapy were all consistent with the guidelines of contemporary oncology.

Therapeutic management in the study group was comprised of primary surgical treatment and neoadjuvant and/or adjuvant therapy. In line with current clinical practice guidelines, patients underwent either breast conservation treatment (BCT) with a sentinel lymph node biopsy procedure or a radical mastectomy, which involved the assessment and, where indicated, the dissection of the axillary lymph node. The choice of treatment modalities and treatment sequencing depended primarily on the clinical stage of BC (local tumor extent, presence of metastases in regional lymph nodes, presence of distant metastases) and the findings of the pathomorphological examination of the tissue samples that were obtained during the biopsy. The pathomorphological protocol also included the stage of malignancy and its molecular features, which were determined by the expression of steroid receptors (ER and PR), the HER2 receptor status and the proliferation rate based on the Ki-67 index. On that basis, algorithms for diagnostic and therapeutic procedures were developed. Neoadjuvant therapy was administered to selected patients with stage IIB HER2-positive breast cancer (trastuzumab). It was administered as chemotherapy to patients with triple-negative cancer and as endocrine therapy (tamoxifen), which was also used after surgery, to patients with hormone receptor-positive cancer (ER, PR).

Additionally, in the period from week six to approximately day 270 after the surgery, the patients received adjuvant therapy in the form of radiotherapy and/or chemotherapy in accordance with the current diagnostic and therapeutic algorithms. Adjuvant radiotherapy was used in the patients with breast-conserving surgery therapy or in selected patients after a mastectomy.

The selection of patients with BC and subjects with benign breast lesions for the study was made on the basis of a gynecological examination. It was followed by an examination by a surgical oncologist or an oncologist, which involved additional tests, i.e., ultrasound, mammography, blood tests and, in selected cases, other imaging tests such as MRI. In all of the study participants (study and control groups), the inflammatory process was excluded by relevant laboratory tests, including a complete blood count, a blood smear, CRP, and enzyme tests. Healthy subjects were selected by a general practitioner and referred to a gynecologist who confirmed their suitability for study participation during a routine checkup at the Gynecology Outpatients Department of the Medical University Hospital in Bialystok. Menopausal status was determined in each subject. All study participants were classified as postmenopausal.

The material used in the study was venous blood that was collected from each study participant into an anticoagulant tube with sodium heparin. The blood was then centrifuged for 15 min at 1000× *g* in order to obtain plasma. The plasma samples were stored at −85 °C until the day of the assay. The VEGF concentrations were measured using the ELISA method with Human VEGF Quantikine Immunoassay provided by R&D Systems Inc., Minneapolis, MN, USA. The ELISA assay was performed according to the manufacturer’s protocols, with duplicate measurements for each standard and sample. This assay employs the quantitative sandwich enzyme immunoassay technique. The intra-assay coefficient of variation (CV%) of VEGF is reported to be 4.5% at a mean concentration of 235 pg/mL (SD = 10.6). The inter-assay CV% of VEGF is reported to be 7.0% at a mean concentration of 250 pg/mL (SD = 17.4). The values of intra- and inter-assay CVs were calculated by the manufacturer and enclosed in the reagent kits. The assay does not exhibit cross-reactivity or interference with numerous human cytokines and other growth factors. The plasma levels of CA 15-3 were measured using the chemiluminescent microparticle immunoassay (CMIA) (Abbott, Chicago, IL, USA) according to the manufacturer’s protocols.

The diagnostic utility of detecting BC recurrence was determined on the basis of the parameters of the mathematical and diagnostic analyses of test results such as diagnostic sensitivity (SE) and specificity (SP), positive and negative predictive value (PPV, NPV), the diagnostic power of the test using the Receiver Operating Characteristics (ROC) curve, and the area under the ROC curve (AUC). The cut-off values were calculated using Youden’s index [20] (as a criterion for selecting the optimum cut-off point) and they were as follows: VEGF was 70.45 pg/mL and CA 15-3 was 18.40 U/mL.

### 2.1. Statistical Analysis

The statistical analysis of the obtained results was performed using the IBM SPSS Statistics 20.0 program (Armonk, NY, USA). Due to a statistically significant deviation from a normal distribution in the distribution of studied quantitative variables in the Shapiro–Wilk test, the differences between the groups were assessed using non-parametric tests. The differences between two groups was assessed using the Mann–Whitney test. When comparing a larger number of groups, the Kruskal–Wallis test was used and supplemented with a post hoc analysis using the Dwass–Steele–Critchlow–Fligner test [21].

Intra-group variations in the concentrations of examined parameters at different time points of the study (prior to and following surgery) were evaluated by the Wilcoxon pairwise test. When making comparisons between a larger number of time points (prior to and following surgery, and during the follow-up), the Friedman test with the Iman–Davenport correction and the post hoc test according to Conover were used. The assessment of the diagnostic power of the tested parameters was based on the analysis of the area under the ROC curve (AUC) using the GraphRoc program (version 1.0) (University of Turku, Turku, Finland) [22].

### 2.2. Ethical Approval

This study was conducted in accordance with the Declaration of Helsinki and the protocol was approved by the local Ethics Committee of the Medical University of Bialystok (R-I-002/239/2015). All of the subjects provided informed consent for study participation.

## 3. Results

### 3.1. Plasma Levels of VEGF in Patients with Breast Cancer before and after Surgery, Classification According to the Tumor Stage

The concentrations of VEGF and CA 15-3 in patients with BC in the preoperative and postoperative periods and during the three-year follow-up are presented in Table 2. The present study demonstrated that in patients with stage I BC, the concentrations of VEGF (121.84 pg/mL) and the reference marker (20.14 U/mL) were statistically significantly higher than in the healthy controls (80.44 pg/mL; 15.94 U/mL; *p =* 0.008; *p* = 0.045, respectively). Moreover, significantly higher preoperative VEGF concentrations were observed in patients with stage I BC in comparison to subjects with benign lesions (58.44 pg/mL; *p* < 0.001). Furthermore, a significant decrease in the plasma VEGF concentration was observed in patients with stage I BC in the postoperative period (week six of observation, 84.48 pg/mL) compared to the level prior to surgery (*p* = 0.016).

It was also found that the preoperative concentrations of VEGF (median: 144.98 pg/mL) and CA 15-3 (23.88 U/mL) in patients with stage II BC were significantly higher in comparison to the healthy controls *(p* = 0.014, *p* = 0.001, respectively). Moreover, significantly higher preoperative VEGF concentrations were observed in patients with stage II BC in comparison to subjects with benign breast lesions (*p* < 0.001). Additionally, a statistically significant decrease in the plasma levels of VEGF, similar to the reduction in CA 15-3 concentration, was observed in patients with stage II BC six weeks after surgery (90.28 pg/mL; 18.84 U/mL, respectively) in comparison to preoperative concentrations (*p* < 0.001; *p* = 0.01).

Based on the statistical analysis of the study results, the preoperative plasma levels of VEGF (128.14 pg/mL) in patients with BC (the total cancer group) were significantly higher than in the healthy controls (*p* = 0.001). The same relationship was observed for the comparative tumor marker CA 15-3 (*p* = 0.001). Moreover, statistically higher VEGF levels were also observed in the total cancer group compared to the benign breast lesions group (*fibroadenoma*) prior to surgery (*p* <0.001). Additionally, a significant postoperative reduction in the plasma VEGF concentration was observed in patients with breast cancer (90.18 pg/mL; *p* = 0.007). The correlation was not observed for CA 15-3.

The same significant reduction in plasma VEGF concentrations as in CA 15-3 (19.6 U/mL) was observed after surgery in the group of subjects with benign breast lesions (40.40 pg/mL; 14.44 U/mL; *p* = 0.023; *p* < 0.001, respectively, for both parameters).

### 3.2. Plasma Levels of VEGF in Patients with Breast Cancer before and after Surgery, Classification According to the Molecular Subtype of BC

The concentrations of VEGF and tumor marker CA 15-3 in patients with BC in all of the study time points are shown in Table 3. Due to a small number of cases of the HER2-positive ER/PR-negative subtype and the triple-negative type of BC, we have excluded those groups from further statistical analysis.

The statistical analysis of the luminal A subtype of BC patients group revealed significantly higher preoperative concentrations of VEGF (142.04 pg/mL) than in the healthy controls (80.44 pg/mL; *p* = 0.035) or in the subjects with benign lesions (58.44 pg/mL; *p* = 0.001). This correlation was not observed for tumor marker CA 15-3. In addition, the postoperative VEGF levels (110.05 pg/mL), as well as the CA 15-3 levels (18.54 U/mL), were significantly reduced compared to their preoperative concentrations (142.04 pg/mL; 20.02 U/mL; *p* < 0.001; *p* = 0.012, respectively).

Based on the analyzed results, the patients with the luminal B subtype of BC had a significantly higher concentration of VEGF (130.55 pg/mL) than the healthy controls (*p* = 0.013) and the subjects with benign lesions (*p* < 0.001). The same tendency was observed for CA 15-3 (23.80 U/mL) in comparison to the healthy controls (*p* = 0.004). The analysis also revealed a significant reduction in both the VEGF and CA 15-3 postoperative levels (93.72 pg/mL; 19.75 U/mL; *p* = 0.001; *p* = 0.017, respectively).

The higher concentrations of CA 15-3 (23.85 U/mL) and VEGF (145.03 pg/mL) were also observed in the group of patients with luminal B HER2-positive BC than in the healthy controls (*p* = 0.006; *p* = 0.001). In contrast to CA 15-3, we also observed a significantly higher VEGF concentration in comparison to the subjects with benign lesions (*p* < 0.001). The analysis of the concentrations of VEGF and CA 15-3 before and after surgery (112.48 pg/mL; 19.40 U/mL) showed significant decreases in the levels of both parameters (*p* = 0.002; in all cases).

No significant differences in the concentrations of the tested parameters were observed between the groups of patients with the selected molecular BC subtypes.

### 3.3. Plasma Levels of VEGF in Patients with Breast Cancer before and after Surgery, Classification According to the Type of Surgery

Table 4 presents the concentrations of VEGF and tumor marker CA 15-3, in all of the time points of the study, in BC patients that were divided into groups according to the type of underwent surgery (BCT or radical mastectomy). The present study demonstrated that in the patients who underwent BCT, the concentration of VEGF (122.45 pg/mL) was significantly higher than in the healthy controls or in the subjects with benign lesions (*p* < 0.001 in all cases). This correlation was not observed for tumor marker CA 15-3 (19.95 U/mL). Furthermore, a significant decrease in the plasma VEGF level was observed for the BCT group in the postoperative period (98.06 pg/mL) compared to the concentration prior to surgery (*p* < 0.001). A similar pre- to postoperative correlation was observed for CA 15-3 (16.50 U/mL; *p* = 0.001).

The same significant reduction in plasma VEGF concentrations was also observed between the patients before radical mastectomy (128.17 pg/mL) and both the healthy controls (*p* = 0.006) and the patients with benign lesions (*p* < 0.001). Additionally, a significant difference was also observed in the CA 15-3 level (23.85 U/mL) between the patients prior to surgery and the healthy controls (*p* < 0.001). There were also significant reductions in both VEGF and CA 15-3 levels compared to their levels before surgery (88.08 pg/mL; 18.05 U/mL; *p* < 0.001; *p* < 0.001, respectively).

### 3.4. Plasma Levels of VEGF in Patients with Breast Cancer after Surgery and Adjuvant Therapy in a Three-Year Follow-Up

#### 3.4.1. Classification According to the Tumor Stage

**Figure 1:**
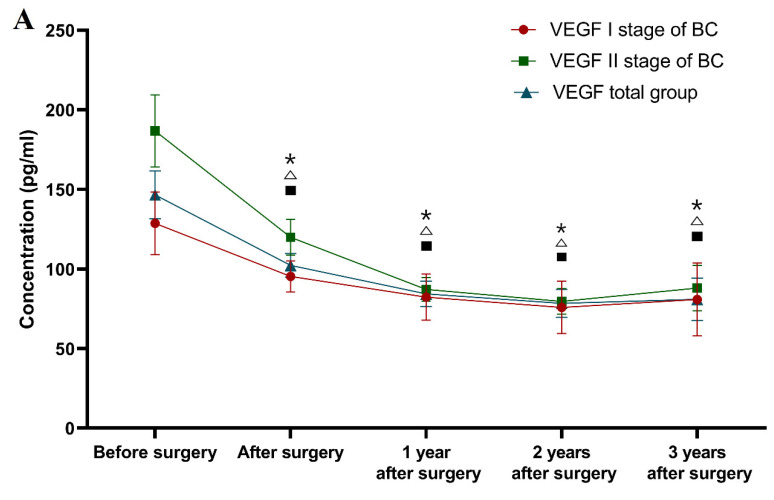

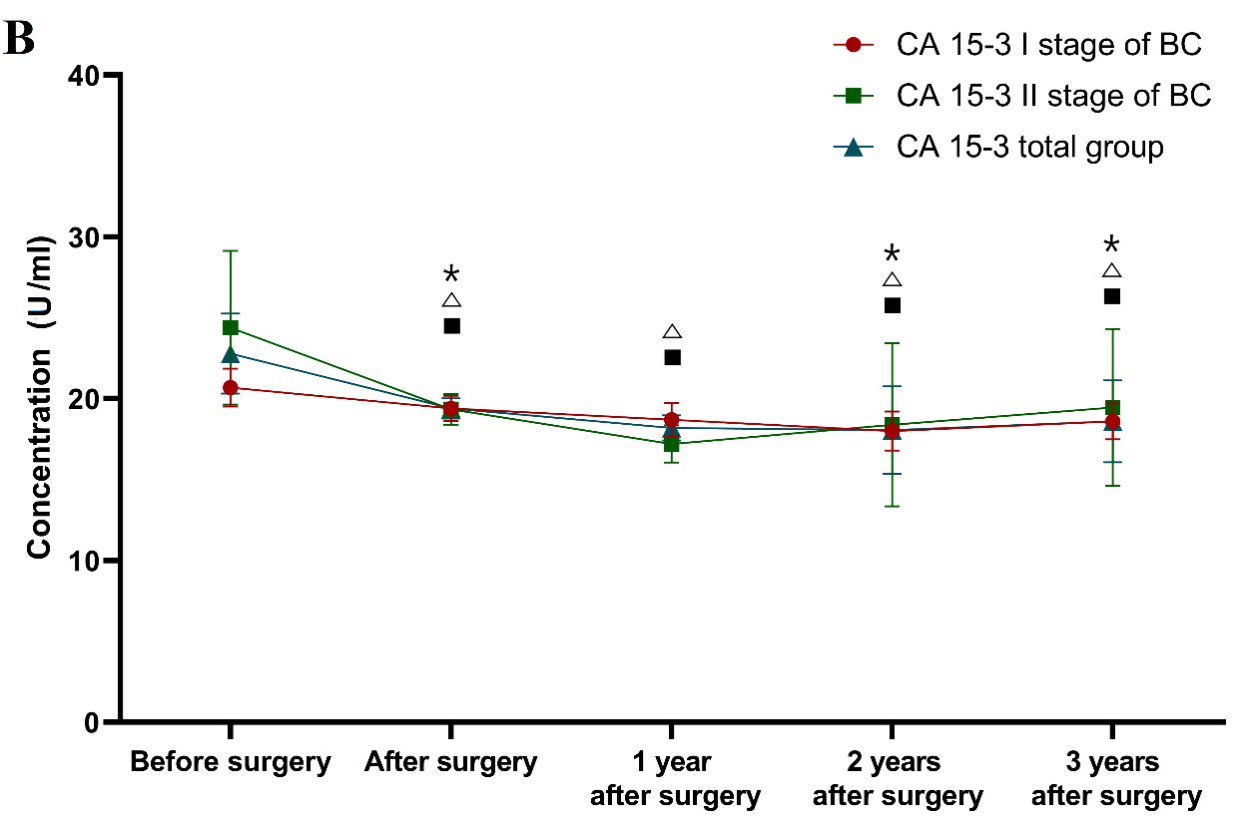
Plasma levels (median and standard error) of VEGF (**A**) and CA 15-3 (**B**) in a three-year follow-up of patients with breast cancer, according to the stage of tumor. * statistically significant difference from preoperative levels in BC stage I group, △ statistically significant difference from preoperative levels in BC stage II group, ▪ statistically significant difference from preoperative levels in BC total group.

Classification according to the tumor stage (see Figure 1).

One year after surgery (and adjuvant therapy), only the CA 15-3 concentration (median 18.44 U/mL) was still significantly higher in the patients with stage I BC in comparison to the healthy controls (*p* = 0.023). The study also demonstrated that the VEGF concentrations one year after surgery were statistically significantly lower (83.24 pg/mL; *p* < 0.001) in comparison to the preoperative levels. Identical relationships regarding VEGF concentrations were found after the second and third years of observation (78.88 pg/mL; 83.44 pg/mL; *p* < 0.001, *p* = 0.001, respectively), and similarly for CA 15-3 (17.84 U/mL; 18.34 U/mL; *p* = 0.001, *p* = 0.002). Surprisingly, the plasma levels of the comparative marker were still significantly higher compared to the healthy controls in the third year after surgery (*p* = 0.012, *p* = 0.011).

During the first, second and third years after surgery, the VEGF concentrations (85.48 pg/mL, 80.34 pg/mL and 81.01 pg/mL) in patients with stage II BC were statistically significantly lower compared to the preoperative levels (*p* = 0.001, *p* < 0.001, *p* = 0.001), which is similar to the CA 15-3 concentrations. After the second and third years of observation, only the CA 15-3 concentration was significantly higher than in the control group (*p* = 0.024, *p* = 0.01).

In the first year after surgery, the VEGF plasma levels (84.72 pg/mL) in the total BC group were statistically significantly lower compared to the preoperative concentrations (*p* < 0.0001), similarly to CA 15-3. The study also revealed that after the second and third years of observation, the VEGF concentrations (79.63 pg/mL; 82.25 pg/mL) were significantly lower in comparison to the preoperative levels (*p* < 0.001 for both periods of observation), identically to CA 15-3 (*p* < 0.001, *p* = 0.001). The concentration of CA 15-3 (18.40 U/mL) was still significantly higher in the first and third years of observation in comparison to the healthy subjects (*p* = 0.02, *p* = 0.01).

#### 3.4.2. Classification According to Molecular Subtype of BC

**Figure 2:**
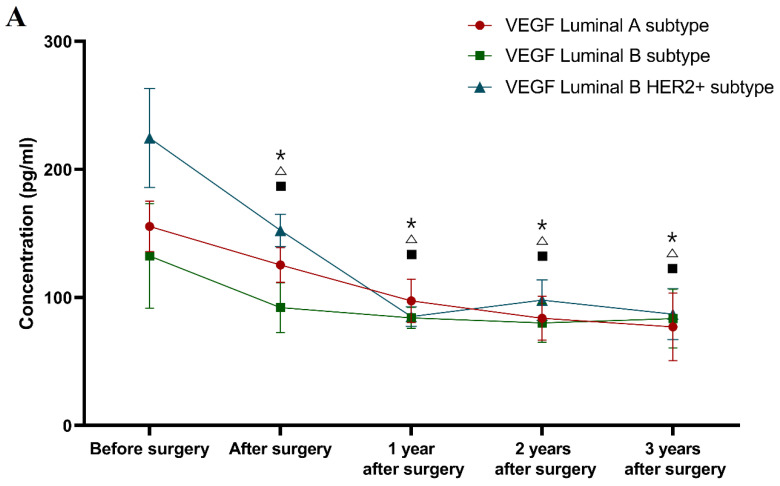

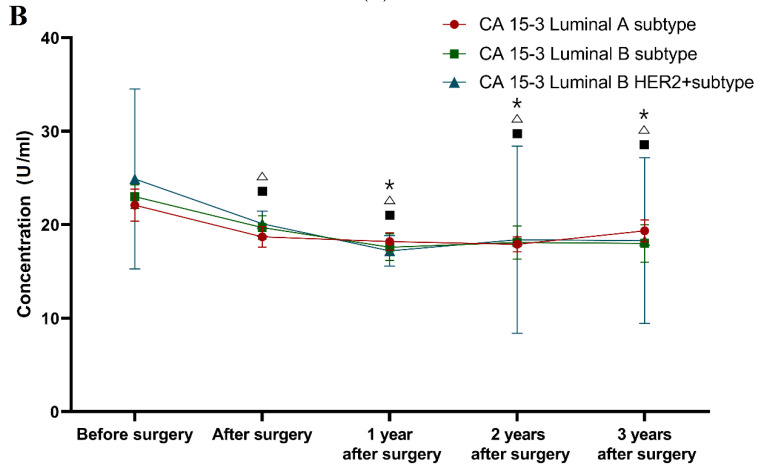
Plasma levels (median and standard error) of VEGF (**A**) and CA 15-3 (**B**) in a three-year follow-up of patients with breast cancer, by the molecular subtype of BC. * statistically significant difference from preoperative levels in luminal A subtype of BC group, △ statistically significant difference from preoperative levels in luminal B subtype of BC group, ▪ statistically significant difference from preoperative levels in luminal B HER2+ subtype of BC group.

Classification according to molecular subtype of BC (see Figure 2).

During the first, second and third years after surgery, the VEGF levels in the patients with all of the selected molecular subtypes of BC (Luminal A subtype 97.34 pg/mL; 83.83 pg/mL; 77.05 pg/mL; Luminal B subtype 84.12 pg/mL; 80.01 pg/mL; 83.49 pg/mL; Luminal B HER2 positive subtype 85.07 pg/mL; 97.98 pg/mL; 87.00 pg/mL) were significantly lower compared to the preoperative concentrations (Luminal A subtype *p* < 0.001; *p* < 0.001; *p* < 0.001; Luminal B subtype *p* = 0.001; *p* < 0.001; *p* = 0.005; Luminal B HER2 positive *p* < 0.001; *p* < 0.001; *p* = 0.001 respectively).

During the three-year follow-up, similar differences as those seen for VEGF were obtained for CA 15-3 (Luminal A subtype 18.20 U/mL; 17.90 U/mL; 19.35 U/mL; *p* = 0.004; *p* = 0.001; *p* = 0.009; Luminal B subtype 17.60 U/mL; 18.10 U/mL; 18.01 U/mL; *p* = 0.028; *p* = 0.005; *p* = 0.021; Luminal B HER2 positive subtype 17.20 U/mL; 18.40 U/mL; 18.30 U/mL; *p* < 0.001; *p* < 0.001; *p* < 0.001 respectively). Moreover, only the comparative marker plasma levels in the luminal A and luminal B subtypes of BC patients were still significantly higher compared to the healthy controls in the third year after surgery (*p* = 0.017, *p* = 0.021).

#### 3.4.3. Classification According to the Type of Underwent Surgery

**Figure 3:**
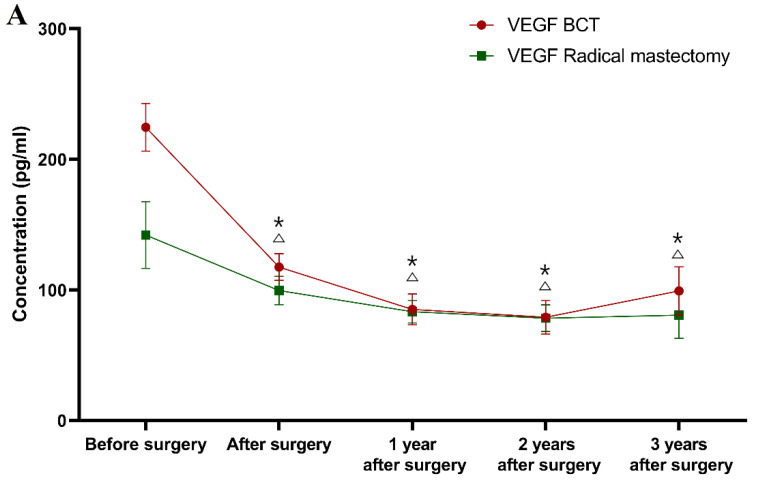

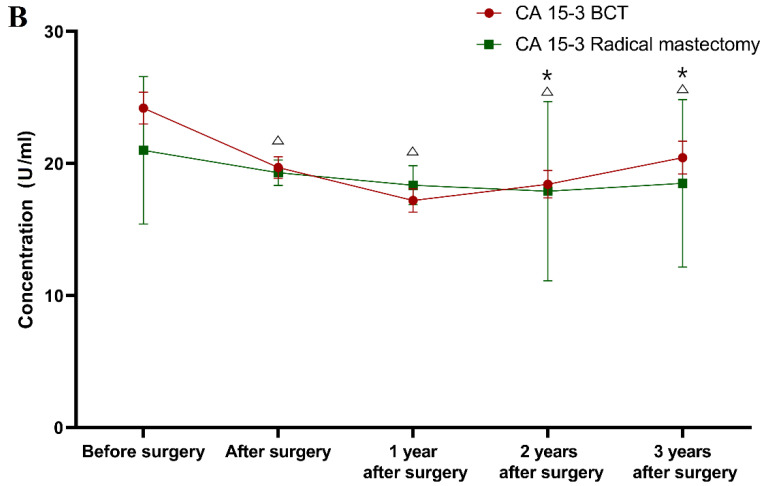
Plasma levels (median and standard error) of VEGF (**A**) and CA 15-3 (**B**) in a three-year follow-up of patients with breast cancer, according to the type of surgery. * statistically significant difference from preoperative levels in BCT group, △ statistically significant difference from preoperative levels in radical mastectomy group.

Classification according to the type of underwent surgery (see Figure 3).

During the first, second and third years after surgery, the VEGF concentrations (83.38 mg/mL; 78.35 mg/mL; 80.64 mg/mL) in the BCT group, as well as in the radical mastectomy group (85.07 pg/mL; 79.16 pg/mL; 99.21 pg/mL) were significantly lower than the concentrations prior to the surgery (BCT group *p* < 0.001 for all of the time points; radical mastectomy group *p* = 0.001; *p* < 0.001; *p* = 0.030).

In the first year after surgery, the CA 15-3 plasma levels (85.07 U/mL) in the radical mastectomy group were statistically significantly lower compared to the preoperative concentrations (*p* < 0.001), in contrast to the BCT group, in which this relationship was not observed. The study also revealed that after the second and third years of observation, the CA 15-3 concentrations (BCT group 17.90 U/mL; 18.50 U/mL; radical mastectomy group 18.42 U/mL; 20.44 U/mL) were still significantly lower in comparison to the preoperative levels in both study groups (BCT group *p* < 0.001; *p* = 0.007; radical mastectomy group *p* = 0.001 for both periods of observation). Similar to the previous results, only CA 15-3 showed significantly higher concentrations in the BCT group in the first and third year of follow-up (*p* = 0.028; *p* = 0.013, respectively) in comparison to the healthy controls, as well as in the radical mastectomy group in the second and third year of follow-up (*p* = 0.020; *p* = 0.012, respectively).

In summary, the greatest dynamics of the concentration changes in patients with BC in a three-year follow-up was demonstrated for VEGF, the plasma levels of which decreased significantly in the total BC group after surgery. Over the period of 1–2 years of observation, in patients with stage I BC, the concentrations of both tested parameters decreased and then marginally increased after the 2–3-year period. The greatest dynamics of concentration changes in the pre- and postoperative periods in patients with stage II BC were demonstrated for VEGF. It should be emphasized that VEGF concentrations, similarly to those of CA 15-3, decreased after surgery. Subsequently, in the period following adjuvant therapy, the plasma levels of VEGF decreased, identically to CA 15-3. During the follow-up period, the VEGF dynamics were identical to those of the stage I BC patients. The difference was shown for CA 15-3, where its level between year 1 and year 2, as well as between year 2 and year 3, only increased (Figure 1).

It should also be highlighted that despite the different divisions into study groups of patients with breast cancer, the dynamics of the changes in the concentrations of the studied parameters were very similar, which can easily be seen by comparing the graphs (Figure 1, Figure 2, and Figure 3).

### 3.5. The Utility of VEGF in the Detection of Breast Cancer Recurrence

Based on the obtained results, recurrence was not detected during the first year of follow-up. In the second year of observation, recurrence was detected in 20 patients (in 4 patients—stage I cancer, in 16 patients—stage II cancer).

**Figure 4:**
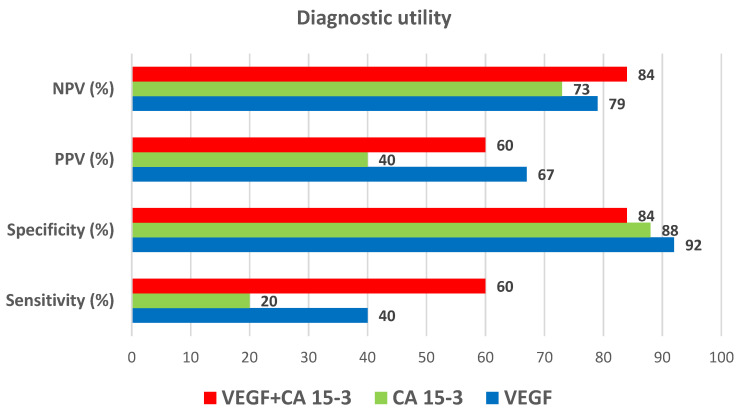
Diagnostic utility of VEGF and CA 15-3 in detection of breast cancer recurrence.

The ability to diagnose BC recurrence on the basis of a positive test result (SE) was highest for VEGF (40%). The combined determination of VEGF and CA 15-3 resulted in an increase in the SE value to 60% (Figure 4).

SP was highest for VEGF (92%) and did not increase when combined with CA 15-3. The probability of detecting BC recurrence based on a PPV reached the highest value for VEGF (67%) and did not increase when combined with the commonly used marker. In addition, high values of probability for excluding BC recurrence were demonstrated based on an NPV, and VEGF again obtained the highest value (79%), which increased in the combined analysis with CA 15-3 to 84% (Figure 4).

**Figure 5:**
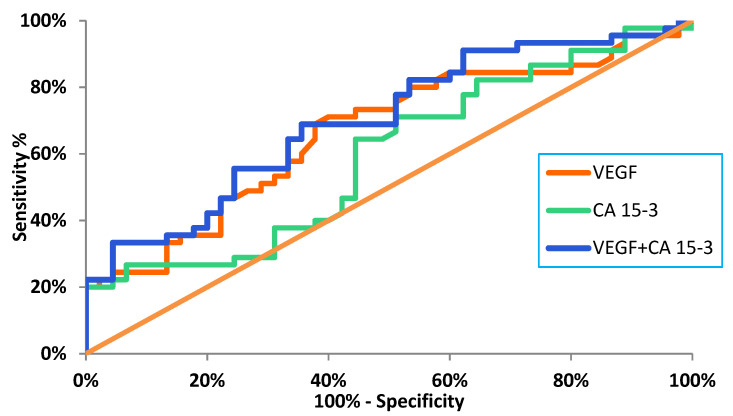
Diagnostic power of tested parameters in the diagnosis of breast cancer recurrence.

The diagnostic power of detecting BC recurrence was highest for VEGF (AUC = 0.6454), which was higher than for CA 15-3 (0.5939), and increased to 0.7074 in the combined analysis of both parameters (Figure 5).

## 4. Discussion

BC is the most common malignancy and accounts for over 30% of all neoplasms diagnosed in women. It is characterized by high, continually increasing morbidity and mortality [1]. Since the currently available modalities of diagnosing BC are imperfect, new diagnostic techniques, including tumor markers, which would enable early detection of this type of cancer, are constantly being sought. At present, search efforts are focused primarily on the identification of new diagnostic parameters that are involved in the neoplastic process, i.e., angiogenesis, lymphangiogenesis, remodeling of the extracellular matrix and degradation of the basal membrane (BM) of endothelial cells. Among them, the molecular markers of carcinogenesis, cytokines, metalloproteinases (MMPs) and their tissue inhibitors (TIMPs) can be distinguished [23,24,25].

Angiogenesis is a multi-step process of forming new blood vessels in which a key role is played by cytokines, particularly VEGF and some MMPs, which act as proteolytic enzymes participating in the degradation of the endothelial cell BM. It has been demonstrated that a high level of these factors (particularly VEGF) significantly contributes to the poor prognosis of patients with BC [26,27,28].

Furthermore, the aim of the present study was to evaluate the concentrations of VEGF and CA 15-3 in the plasma of patients with low-stage BC, as well as in control groups, i.e., subjects with benign breast lesions (*fibroadenoma*), and healthy individuals. Moreover, the utility of the studied parameters in the assessment of the long-term efficacy of the surgical removal of the tumor, as well as in the detection of recurrence, was evaluated. It seemed to us a worthwhile idea to create a new tumor marker that would be useful in the diagnosis of BC, as well as serving as a prognostic parameter.

The statistical analysis of the obtained test results revealed that the preoperative plasma concentrations of VEGF and the comparative marker CA 15-3 in patients with BC were significantly higher than the concentrations in the control group of healthy women. Similar results have been obtained in our earlier studies [14,15,16,28] and in the studies by other authors [17,29]. A study by Quaranta et al. [30] failed to demonstrate a statistically higher VEGF concentration in comparison to healthy subjects, although only 20 women constituted the control group. Moreover, Jovino et al. [31] observed a positive correlation between the serum levels of VEGF and tissue expression, which indicates the role of this cytokine in the pathogenesis of BC.

In the present study, statistically significantly higher VEGF concentrations were observed in the total group of patients with BC compared to the control group with benign lesions (*fibroadenoma*) in the preoperative period. No such correlation was observed for CA 15-3. Regarding VEGF, similar results were obtained by Xu et al. [32], whose study nevertheless compared only 45 patients with BC to 16 subjects with benign lesions, and by Salven et al. [33]. However, the study groups in both investigations were composed of patients with various histological types of BC. The observations have been confirmed by Ławicki et al. in a larger study group and in subjects with benign lesions [14,28], and by other authors [17].

Moreover, a significant decrease in the VEGF plasma levels was observed in the patients with BC (the entire study group) and the subjects with benign lesions following surgery, compared to its preoperative status. An identical relationship was found for CA 15-3. The results are consistent with those reported by Jing et al. [34], who observed a decrease in VEGF concentration not only immediately after surgery, but also 120 days following surgery. Similar results were obtained by Findeisen et al. [35]. Contradictory findings were reported by Rocca et al. [36] who did not find any differences between pre- and postoperative VEGF concentrations, although the authors studied the serum of patients with BC.

The present study indicates the utility of VEGF in the assessment of the long-term efficacy of the surgical removal of BC as well as the effectiveness of adjuvant therapy. Our observations are consistent with studies by other authors in the BC cell model, in which the absence of a decrease in VEGF concentration or its increase following surgery indicated the incomplete removal of the neoplastically transformed cells [35,37].

This study demonstrated that the preoperative concentrations of VEGF and CA 15-3 in patients with stage I BC were statistically higher than in the control group of healthy subjects. Moreover, significantly higher preoperative VEGF concentrations were observed in patients with stage I BC compared to the control group of subjects with fibroadenoma, with no comparable correlation observed for CA 15-3. Similar relationships have previously been reported by Ławicki et al. [14,28]. In the present study, we also observed significant differences in VEGF concentrations between the healthy controls and the subjects with benign lesions. Interestingly, CA 15-3 did not show similar dependencies. In patients with stage II BC, the preoperative VEGF concentrations were statistically significantly higher compared to both control groups. The comparative marker (CA 15-3) showed significantly higher concentrations only in relation to the group of healthy women. Differences between our results and those that were presented in other research papers may be explained by a different composition and selection of study and control groups, as well as a different number of participants [14,16,28].

As for evaluating the applicability of the tested parameters to detect BC recurrence, it was revealed that the ability to diagnose BC on the basis of a positive test result (SE) was highest for VEGF (40%). It was higher than for the routinely used marker CA 15-3. Due to a lack of available literature reports, it was not possible to compare the results of the present study with those that were obtained by other authors. The most important finding was that VEGF showed high values of probability for excluding BC recurrence solely on the basis on a negative test result (NPV). The diagnostic power of detecting BC recurrence was also highest for VEGF (AUC = 0.6652), which was higher than for CA 15-3 (0.5941), and the values increased significantly (0.7074) in the combined analysis of both parameters. This approach to the research topic is very innovative since no similar studies regarding VEGF in the detection of cancer recurrence have been found in the available literature. In a study by Zajkowska et al., in which the diagnostic potential of selected parameters (including VEGF) in BC was also evaluated, the highest diagnostic power was demonstrated for VEGF in patients before surgery [38].

The present study revealed that VEGF does not play a significant role in monitoring the effectiveness of adjuvant therapy for BC (although this was not the aim of the present investigation). We were not able to discuss our results regarding the changes in the concentrations of parameters tested, depending on the type of surgery or the molecular BC subtype, during the three-years observation period due to the lack of papers demonstrating similar data.

In summary, the obtained results suggest the diagnostic utility of VEGF in the evaluation of the efficacy of the surgical treatment of BC as well as in the detection of BC recurrence, but only in the combined analysis with the routinely used marker CA 15-3 as a new diagnostic panel.

## 5. Conclusions

Our study indicates the diagnostic utility of VEGF in BC and its role as a prognostic marker in the evaluation of the efficacy of the surgical removal of BC and in the detection of recurrence, which is particularly suggested by the combined analysis with CA 15-3 as a new diagnostic tumor marker panel.

## Figures and Tables

**Table 1:** Characteristics of study and control groups.

Groups		Median Age	Number of Patients
		Range	
**Breast cancer group**	stage I T_1_N_0_M_0_—50 patients	54 49–70	50
	stage II IIA—T_2_N_0_M_0_—8, IIB: T_2_N_1_M_0_—23, T_3_N_0_M_0_—19	53 50–70	50
	Total group (I + II stage)	53 49–70	100
	Luminal A subtype	52 49–60	34
	Luminal B subtype	53 50–62	24
	Luminal B HER 2 positive subtype	51 49–57	24
	HER 2 positive ER/PR negative subtype	53 50–70	11
	Triple negative type	54 50–64	7
	Menopausal status	All women were postmenopausal	
**Control groups**	Benign breast tumor subjects (*fibroadenoma*)	51 47–60	50
	Healthy subjects	51 48–62	50
	Menopausal status	All women were postmenopausal	

**Table 2:** Plasma levels of tested parameters in a three-year follow-up, groups according to the tumor stage of BC.

	Before Surgery	Six Weeks after Surgery	One Year after Surgery	Two Years after Surgery	Three Years after Surgery
	**Breast cancer—stage I**				
**VEGF pg/mL**	**^1,2,3^**		**^4^**	**^4^**	**^4^**
Median	121.84	84.48	83.24	78.88	83.44
Range	26.56–700.00	8.42–422.38	20.94–422.16	31.01–348.09	32.9–490.84
**CA 15-3 U/mL**	**^1^**		**^1^**	**^4^**	**^1,4^**
Mediana	20.14	18.54	18.44	17.84	18.34
Range	6.2–49.40	4.70–29.60	12.90–35.60	10.10–44.25	14.20–37.60
	**Breast cancer—stage II**				
**VEGF pg/mL**	**^1,2,3^**		**^4^**	**^4^**	**^4^**
Median	144.98	90.28	85.48	80.34	81.01
Range	18.63–759.38	13.31–405.17	23.62–208.23	23.62–208.22	32.12–398.44
**CA 15-3 U/mL**	**^1,3^**		**^4^**	**^1,4^**	**^1,4^**
Median	23.88	18.84	17.22	18.48	21.54
Range	4.4–250.25	7.20–40.50	5.10–36.90	7.10–167.50	11.70–150.40
	**Breast cancer—total group**				
**VEGF pg/mL**	**^1,2,3^**		**^4^**	**^4^**	**^4^**
Median	128.14	90.18	84.72	79.63	82.25
Range	18.63–759.38	8.42–422.38	23.62–422.16	23.62–348.09	32.12–490.84
**CA 15-3 U/mL**	**^1,3^**		**^1,4^**	**^4^**	**^1,4^**
Median	21.40	18.40	18.15	18.00	19.15
Range	4.8–240.25	4.70–40.50	5.10–36.90	7.10–167.50	11.70–150.40
	***Fibroadenoma***				
**VEGF pg/mL**	**^3^**		**^-^**		
Median	58.44	40.40			
Range	14.06–200.75	14.00–227.30			
**CA 15-3 U/mL**	**^3^**		**^-^**		
Median	19.64	14.44			
Range	7.50–45.40	7.00–30.02			
	**Healthy subjects**				
**VEGF pg/mL**					
Median	80.44				
Range	20.63–477.92				
**CA 15-3 U/mL**					
Median	15.94				
Range	6.60–27.20				

**^1^** Statistically significant differences between patients with breast cancer and healthy controls; **^2^** Statistically significant differences between patients with breast cancer and subjects with benign breast lesions before surgery (*fibroadenoma*); **^3^** Statistically significant differences between preoperative and postoperative concentrations in patients with breast cancer or subjects with benign lesions; **^4^** Statistically significant differences between concentrations before surgery and one, two and three years after surgery in patients with breast cancer.

**Table 3:** Plasma levels of tested parameters in a three-year follow-up, groups by the molecular subtype of BC.

	Before Surgery	Six Weeks after Surgery	One Year after Surgery	Two Years after Surgery	Three Years after Surgery
	**Luminal A subtype**				
**VEGF pg/mL**	**^1,2,3^**		**^4^**	**^4^**	**^4^**
Median	142.04	110.05	97.34	83.83	77.05
Range	18.63–423.19	8.42–422.38	19.58–422.16	31.01–348.09	32.12–490.84
**CA 15-3 U/mL**	**^3^**		**^4^**	**^4^**	**^1,4^**
Mediana	20.02	18.54	18.20	17.90	19.35
Range	4.60–49.40	7.10–40.50	11.90–34.60	10.10–30.10	11.70–37.60
	**Luminal B subtype**				
**VEGF pg/mL**	**^1,2,3^**		**^4^**	**^4^**	**^4^**
Median	130.55	93.72	84.12	80.01	83.49
Range	23.19–750.60	20.94–405.17	20.55–186.83	23.62–290.60	40.10–398.44
**CA 15-3 U/mL**	**^1,3^**		**^4^**	**^4^**	**^4^**
Median	23.80	19.75	17.60	18.10	18.01
Range	12.10–36.30	4.70–31.50	5.10–34.30	7.10–44.25	12.90–48.10
	**Luminal B HER2 positive subtype**				
**VEGF pg/mL**	**^1,2,3^**		**^4^**	**^4^**	**^4^**
Median	145.03	112.48	85.07	97.98	87.00
Range	46.23–759.38	23.76–242.31	32.12–136.21	44.05–290.60	35.23–345.22
**CA 15-3 U/mL**	**^1,3^**		**^4^**	**^4^**	**^1,4^**
Median	23.85	19.40	17.20	18.40	18.30
Range	4.40–250.25	7.20–39.60	12.50–36.90	7.10–167.50	12.90–150.40

**^1^** Statistically significant differences between patients with breast cancer and healthy controls; **^2^** Statistically significant differences between patients with breast cancer and subjects with benign breast lesions before surgery (*fibroadenoma*); **^3^** Statistically significant differences between preoperative and postoperative concentrations in patients with breast cancer or subjects with benign lesions; **^4^** Statistically significant differences between concentrations before surgery and one, two and three years after surgery in patients with breast cancer.

**Table 4:** Plasma levels of tested parameters in a three-year follow-up, groups by the type of undergone surgery.

	Before Surgery	Six Weeks after Surgery	One Year after Surgery	Two Years after Surgery	Three Years after Surgery
	**Breast conserving therapy (BCT)**				
**VEGF pg/mL**	**^1,2,3^**		**^4^**	**^4^**	**^4^**
Median	122.45	98.06	83.38	78.35	80.64
Range	18.63–700.00	8.42–422.38	19.58–422.16	31.01–348.09	32.12–490.84
**CA 15-3 U/mL**	**^3^**		**^1^**	**^4^**	**^1,4^**
Mediana	19.95	16.50	18.35	17.90	18.50
Range	4.60–51.30	6.60–27.20	11.80–35.60	7.10–44.25	14.20–48.10
	**Radical mastectomy**				
**VEGF pg/mL**	**^1,2,3^**		**^4^**	**^4^**	**^4^**
Median	128.17	88.08	85.07	79.16	99.21
Range	23.19–759.38	13.31–367.65	20.55–186.83	23.62–208.22	35.23–398.44
**CA 15-3 U/mL**	**^1,3^**		**^4^**	**^1,4^**	**^1,4^**
Median	23.85	18.05	17.20	18.42	20.44
Range	4.40–250.25	9.10–39.60	5.10–36.90	14.10–167.50	11.70–150.40

**^1^** Statistically significant differences between patients with breast cancer and healthy controls; **^2^** Statistically significant differences between patients with breast cancer and subjects with benign breast lesions before surgery (*fibroadenoma*); **^3^** Statistically significant differences between preoperative and postoperative concentrations in patients with breast cancer or subjects with benign lesions; **^4^** Statistically significant differences between concentrations before surgery and one, two and three years after surgery in patients with breast cancer.

## Data Availability

The data presented in this study are available on request from the corresponding author.
